# A coevolution analysis for identifying protein-protein interactions by Fourier transform

**DOI:** 10.1371/journal.pone.0174862

**Published:** 2017-04-21

**Authors:** Changchuan Yin, Stephen S. -T. Yau

**Affiliations:** 1 Department of Mathematics, Statistics and Computer Science, The University of Illinois at Chicago, Chicago, IL 60607-7045, United States of America; 2 Department of Mathematical Sciences, Tsinghua University, Beijing 100084, China; Huazhong University of Science and Technology, CHINA

## Abstract

Protein-protein interactions (PPIs) play key roles in life processes, such as signal transduction, transcription regulations, and immune response, etc. Identification of PPIs enables better understanding of the functional networks within a cell. Common experimental methods for identifying PPIs are time consuming and expensive. However, recent developments in computational approaches for inferring PPIs from protein sequences based on coevolution theory avoid these problems. In the coevolution theory model, interacted proteins may show coevolutionary mutations and have similar phylogenetic trees. The existing coevolution methods depend on multiple sequence alignments (MSA); however, the MSA-based coevolution methods often produce high false positive interactions. In this paper, we present a computational method using an alignment-free approach to accurately detect PPIs and reduce false positives. In the method, protein sequences are numerically represented by biochemical properties of amino acids, which reflect the structural and functional differences of proteins. Fourier transform is applied to the numerical representation of protein sequences to capture the dissimilarities of protein sequences in biophysical context. The method is assessed for predicting PPIs in Ebola virus. The results indicate strong coevolution between the protein pairs (NP-VP24, NP-VP30, NP-VP40, VP24-VP30, VP24-VP40, and VP30-VP40). The method is also validated for PPIs in influenza and *E.coli* genomes. Since our method can reduce false positive and increase the specificity of PPI prediction, it offers an effective tool to understand mechanisms of disease pathogens and find potential targets for drug design. The Python programs in this study are available to public at URL (https://github.com/cyinbox/PPI).

## 1 Introduction

Proteins are essential molecules in all biological systems in a cell, with most proteins requiring protein-protein interactions (PPIs) to function effectively. For example, transport proteins interact with structural proteins and hormone peptides interact with receptors. Some proteins form structural complexes, and the interactions among different protein complexes are necessary for cell functions. Protein interactions are fundamentally characterized as stable or transient, and both types of interactions can be either strong or weak. If two protein interact via physical contact and the affinity is strong, the strong interaction can be detected using in-vitro biochemical experiments such as pulling-down and co-immunoprecipitation assays. However, biochemical experiments for PPIs are time-consuming and expensive, making it difficult to study complete protein interaction networks within a genome [1].

Recently, computational methods for detecting PPIs based on coevolution analysis have distinguished themselves from biochemical experiments and other computational methods [2, 3]. Protein evolution is the result of natural selections of mutations that have functional advantages over other random mutations. The interactions of proteins from coevolution can be maintained by either direct binding or functional association. If two proteins interact with each other, when one protein undergoes a mutation, the other protein may have a compensatory mutation, otherwise, the two proteins cannot maintain the stability or functions of the interaction over the course of evolution. Evolutionary pressure thus creates coevolution pairs of proteins in cells that maintain the PPI. Two phylogenetic trees constructed by two interacted proteins through MSA are expected to be similar and the detection of significant correlations of phylogenetic trees is used to infer probable coevolution and interactions [4, 5]. However, due to the intrinsic nature of phylogenetic trees in related organisms, existing coevolution analysis methods that are based on sequence alignments usually have high false positive rates [6, 7].

To address the problems in MSA based coevolution method, several new feature encoding and extraction methods in PPI predictions have been developed. Converse vectors encoding of protein sequence pairs based on k-mer scheme can improve the accuracy of PPI prediction [8]. The geometrical feature representation for the similarity measure of proteins are also important to predict PPIs [9]. Sequence features from covariations at coevolving positions may improve the performance of PPI prediction [10]. However, these feature representations do not include the biochemical properties of amino acids in position context.

We present here a novel alignment-free method for coevolution analysis. The method is based on biochemical properties of amino acids, instead of using sequence alignments. Comparison of sequence similarity adopts discrete Fourier transform (DFT) as an analysis method. Using coevolution analysis, we apply this DFT method to investigate the interactions of all seven proteins in Ebola virus.

Ebola virus is a filamentous, nonsegmented, negative-strand RNA virus. Ebola virus infects both primates and humans and leads to severe hemorrhagic fever, with high mortality rates. Understanding the PPIs of Ebola virus will advance the development of effective vaccines. Ebola virus genome encodes seven proteins: glycoprotein (GP), nucleoprotein (NP), RNA polymerase (L), VP24, VP30, VP35, and VP40 [11]. Glycoprotein (GP) is the major spike surface protein and enables virus to attach and entry to host. VP40 is the most abundant virion protein, and works as major matrix protein in assembly and budding of Ebola virus. VP24 is minor matrix proteins of mature virions. Three proteins GP, VP40 and VP24 build up the multi-layered virus envelope. Four proteins NP, VP35, VP30, and L form the ribonucleoprotein complex that mediates transcription and replication of the viral genome.

We perform a case study on the effectiveness of the DFT method on PPI predication. The study on PPIs in Ebola virus and influenza virus using DFT based coevolution analysis indicates that our method is accurate, effective and thus outperforms the coevolution methods that are based on sequence alignments. Our approach significantly contributes to the PPI analysis of proteins in various genomes.

## 2 Methods and algorithms

We present an effective computational method to identify PPIs based on the coevolution model. The method employs chemical properties of proteins in phylogenetic analysis. The method uses the Euclidean distance of Fourier transform of proteins as dissimilarity measure for pairwise protein interactions. To predict if two proteins A and B interact in a species by coevolution analysis, we first collect the protein sequence mutations in a collection of species. The two proteins may undergo different mutations in these genomes. We compute the distance matrix of protein A using DFT distance measure. Similarly, we get the distance matrix of protein B in the same set of genomes. We then compute the correlation of two distance matrices A and B. If two proteins A and B interact, the distance matrices A and B have high correlation, otherwise, the correlation is low. Finally, we use multidimensional scaling (MDS) to visualize the correlation between the matrices as a measure for the interaction distance.

### 2.1 Representation of protein sequences by hydrophobicity properties of amino acids

The existing methods in coevolution analysis for PPI are based on multiple sequence alignments by sequence characters. Yet character based similarity cannot provide insight into the structural aspects of a protein. The biological function of a protein is the direct consequence of its sequence and is determined by the chemical properties of the sequence. There are hundreds of physico-chemical properties in 20 amino acids of proteins [12]. Examples of important physico-chemical properties are the hydrophobicity, polarizability, Van der Walls volume, ionization constant, accessible solvent surface area, etc. Among all of these physico-chemical properties, hydrophobicity property is of importance for protein structure folding and thus determines protein-protein interaction. Hydrophobicity properties of amino acids have been used as an efficient way to compare and analyze amino acid sequences [7].

Comparison of protein sequences for PPI shall consider the contributions of physicochemical properties, but none of the physico-chemical properties has been employed in existing PPI method by coevolution analysis. To understand the conservation of residues in protein sequences during coevolution, it is important to qualitatively and quantitatively measure the differences among residues using physico-chemical properties of amino acids. The functions of a protein depend on how the protein folds into 3D structure which most importantly depends on the hydrophobicity properties of the proteins. Therefore, effective PPI prediction methods need to capture hydrophobicity properties of the proteins. In this study, we use the hydrophobicity values to represent protein sequences (Table 1).

**Table 1:** Kyte-Doolittle hydrophobicity values for 20 amino acids [13].

amino acid	hydrophobicity	amino acid	hydrophobicity
A	1.8	M	1.9
C	2.5	N	-3.5
D	-3.5	P	-1.6
E	-3.5	Q	-3.5
F	2.8	R	-4.5
G	-0.4	S	-0.8
H	-3.2	T	-0.7
I	4.5	V	4.2
K	-3.9	W	-0.9
L	3.8	Y	-1.3

### 2.2 Constructing distance matrix of proteins by Fourier transform

**Fig 1:**
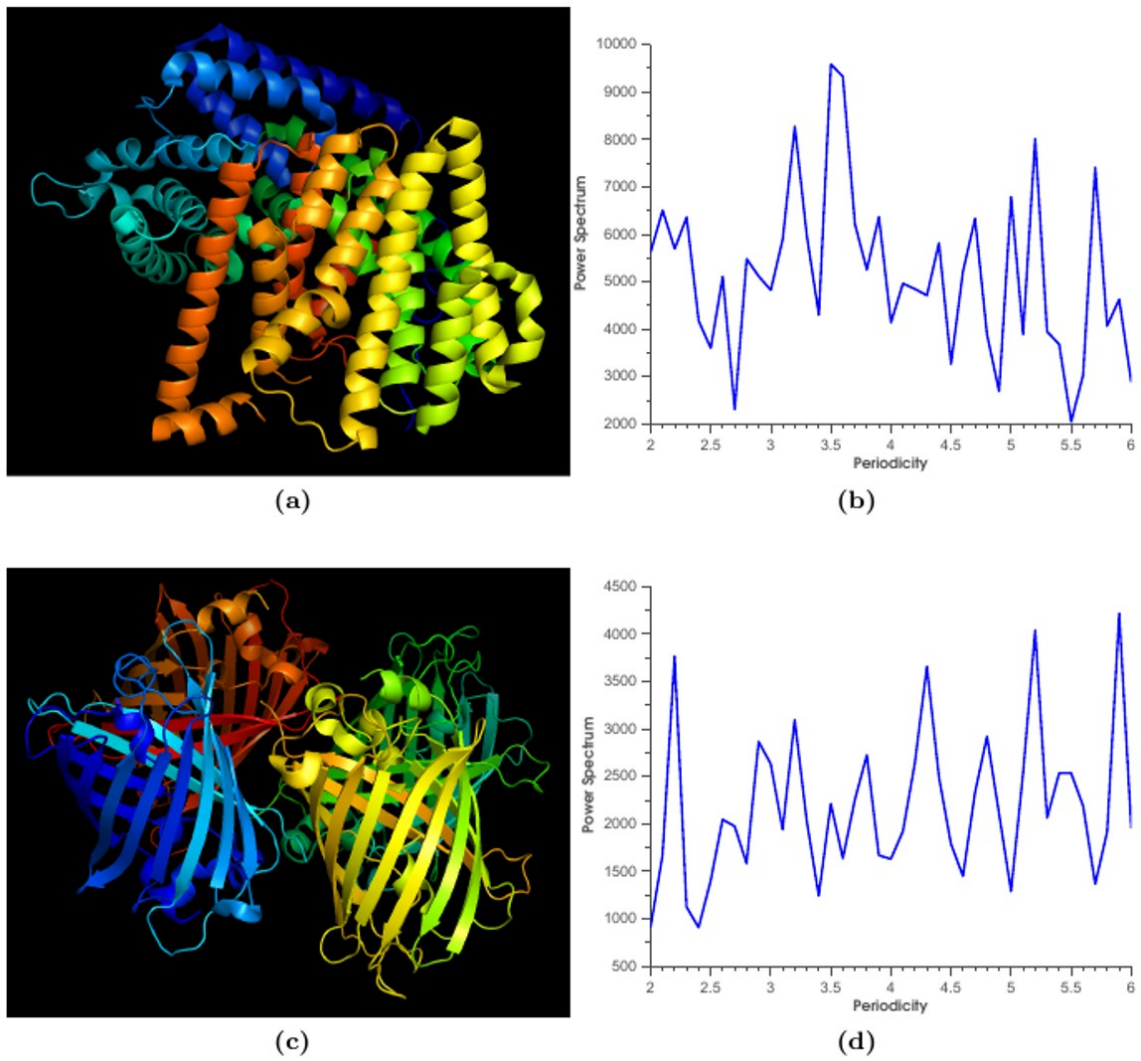
Fourier transform analysis of two protein sequences. (a) Crystal structure of sesquiterpene synthase (PDB:4GAX). (b) Fourier power spectrum of sesquiterpene synthases. (c) Crystal structure of green fluorescent protein (PDB:1W7T). (d) Fourier power spectrum of green fluorescent protein.

Distance between protein sequences can be measured through Fourier transform of protein hydrophobicity profiles. Discrete Fourier transform (DFT), a broadly used digital signal processing approach, transforms data from time space to frequency space and reveals periodicities that are hidden in time space. Fourier transform gives a unique representation of the original underlying signal in frequency domain. The frequency domain vector contains all the information about signal in time domain. To analyze the distribution of hydrophobicity along protein sequence positions, a sliding window is used to extract different length protein sequence segment and then apply DFT on each windowed protein sequence. we performed Fourier transform of the sequence of sesquiterpene synthases from *Artemisiaannua*, which contains *α*-helix structures (Fig 1(a)). The protein sequence was first converted to numerical vector of Kyte-Doolittle hydrophobicity values. The DFT analysis shows periodicity-3.6 of *α*-helix structures in the protein sequence (Fig 1(b)). The periodicity-3.6 of hydrophobicity reflects the period of 3.6 polar and nonpolar residues of an *α*-helix structure [14]. The other example is Fourier analysis of the sequence of green fluorescent protein (PDB:1W7T), which contains *β*-sheet structures (Fig 1(c)). The DFT analysis shows periodicity-2.3 of *β*-sheet structures in the protein sequence (Fig 1(d)). The periodicity-2.3 of hydrophobicity reflects the periodic arrangement of 2.3 polar and nonpolar residues in a *β*-sheet structure [14]. These two examples show that Fourier power spectrum of hydrophobicity vector may infer the structural features of protein because hydrophobicity is the major driven force for protein folding. The DFT method has been extensively used to study periodicities and repetitive elements in genomes and protein structures [15, 16]. Moreover, we previously use DFT to predict protein coding regions [17, 18], and compare similarities of DNA sequences [19–21]. Thus we may reply on Fourier transform to accurately compare protein sequences in coevolution analysis of PPIs. Let *X*(*k*) be the DFT of time series *x*(*n*) of length *N*, and *X*(*k*) is defined as [22]

$$X\left(k\right)=\sum_{n=0}^{N-1}x\left(n\right)e^{-i\frac{2\pi }{N}kn},k=0,1,2,\cdots ,N-1$$ (1)

where $i=\sqrt{-1}$.

We can infer the information content in protein sequences from the distribution of Fourier coefficients because the original sequence can be recovered from the Fourier coefficients by inverse Fourier transform. The relationship between original sequence and its Fourier transform is one-to-one, therefore, the DFT method can be efficient and effective in comparing similarities of protein sequence mutations in coevolution analysis. We define the distance between two time series *a* and *b* by the corresponding Fourier transform coefficients *A* and *B* as follows

$$dist\left(A,B\right)=\sqrt{\sum_{i=0}^{N-1}{\left(\text{R}\left(A\left(i\right)\right)-\text{R}\left(B\left(i\right)\right)\right)}^{2}+\sum_{i=0}^{N-1}\left(\text{I(A}\left(i\right)\right)-{\text{I(B}\left(i\right)))}^{2}}$$ (2)

where *R*(*A*(*i*)) and *I*(*A*(*i*)) are the real part and the imaginary part of complex number *A*(*i*), respectively.

When two protein sequences *x*(*t*) and *y*(*t*) are of different lengths, the DFT of the two sequences have different lengths, the Euclidean distance of DFT power spectrum or DFT coefficients of protein sequences of unequal lengths cannot be directly computed. Some solutions to this problem in the signal processing are to extend the shorter series *y*_*t*_, by padding zeros or even scaling method to extend power spectra of short lengths to the longest one [20]. We use padding zeros approach in this study. The example of Fourier transform analysis of VP24 protein of Ebola virus is provided in supplementary materials (S1 File).

After Fourier transform of numerical sequences of proteins from different species, we can get pairwise distance matrix of different species based on a specific protein and construct the phylogenetic tree of these species. Our algorithm for computing the pairwise distance of two genomes from a protein is as follow.

**Algorithm 1:** Computing pairwise Euclidean distances of genomes by their specific protein sequences in Fourier frequency domain.

**Data:** a set of genome, protein X

**Result:** distance of genome a and b

Steps

1. Convert protein sequences X in genome a and b into numerical series using hydrophobicity values of amino acids sequences.
2. For all the numerical sequences, pad zeros to extend numerical sequences the longest length in the genome set.
3. Apply DFT to numerical sequences to frequency domain and get Fourier transform of the sequence.
4. Construct distance matrix from the pairwise Euclidean distances.
5. Construct phylogenetic tree of the genome set from the distance matrix.

Our algorithm for identifying interaction between proteins X and Y is as follows.

**Algorithm 2:** Identifying interaction between proteins X and Y.

**Data:** Protein X and Y, a set of genomes

**Result:** Co-evolution distance of protein X and Y

Steps

1. Construct distance matrix X of the genomes by protein X (Algorithm 1).
2. Construct distance matrix Y of the genomes by protein Y (Algorithm 1).
3. Compute correlation coefficients of distance matrices X and Y.
4. Visualize the interaction distance of protein X and Y from distance correlations by multidimensional scaling analysis (MDS).

### 2.3 Correlation of two proteins in coevolution analysis

To detect if two proteins X and Y interact with each other, we first collect protein sequences X and Y from the same set of genomes, we then use the proposed DFT method to construct the distance matrices for protein X and Y, respectively. The correlation between two distance matrices is used to indicate if two protein X and Y interact. The correlation of protein mutations in an interaction pair is measured by the Pearson correlation *cor* of two distance matrices as follows.

$$cor=\frac{\sum_{i=1}^{n}\left(X_{i}-\bar{X}\right)(Y_{i}-\bar{Y})}{\sqrt{\sum_{i=1}^{n}{\left(X_{i}-\bar{X}\right)}^{2}}\sqrt{\sum_{i=1}^{n}{\left(Y_{i}-\bar{Y}\right)}^{2}}}$$ (3)

where *n* is the number of elements of the upper triangular of the distance matrices, $\bar{X}$ and $\bar{Y}$ are means of *X* and *Y*, respectively. For a set of *N* proteins, these pairwise Pearson correlations form a correlation matrix *C* ∈ *R*^*N* × *N*^.

To increase the specificity of PPI method, the genomes with a pair of normal proteins are excluded in the coevolution analysis. In details, if each of two proteins X and Y has no mutation in genomes A and B, the DFT distance of protein sequence X (or Y) between two genomes A and B is zero. These two genomes A and B are excluded in coefficient computation.

### 2.4 Co-evolution analysis by MirrorTree

One widely used computational method for inferring PPI is coevolution analysis of MirrorTree from MSA [2, 23]. To detect if two proteins X and Y interact, the Mirrortree method first retrieves the protein sequence X (or Y) from a set of genomes, then performs the multiple sequence alignment of the protein sequences X (or Y). For the PPI analysis in Ebola virus by the MirrorTree method, we first extract all protein sequences from 75 Ebola virus genomes. The GenBank access numbers of the virus genomes are listed in supplementary materials (S2 File). We align each pair proteins among all these virus species by Cluster Omega (http://www.ebi.ac.uk/Tools/msa/) [24], and the aligned sequence files are used as inputs for the MirrorTree server to construct MirrorTree and compute coevolution correlation coefficients (http://csbg.cnb.csic.es/mtserver/) [25].

### 2.5 Multidimensional scaling analysis (MDS)

We employ MDS method to visualize the relative interaction distance of PPIs in two-dimensions. Multidimensional scaling (MDS) projects a distance matrix into a set of coordinates such that the Euclidean distances of these coordinates approximate the original distances [26]. Since the correlation of proteins from coevolution actually measures the similarity of corresponding protein mutations, the Pearson correlation matrix *C* is first transformed to 1 − *C* as dissimilarity distance matrix *D*, which represents relative interactions of proteins.

The MDS method is then applied to the dissimilarity distance matrix *D*. The MDS analysis for visualization is as follows. From the distance matrix *D* of *N* protein samples, we construct the Gram matrix B as $B=-\frac{1}{2}\left(HDH\right)$, where $H=I-\frac{1}{N}ee^{T}$, *I* ∈ *R*^*N* × *N*^ is the identity matrix and *e* ∈ *R*^*N* × *N*^ is a vector of all ones. The matrix B has maximum r non-zeros eigenvalues and *B* = *VΛV*^*T*^, if we take *r* largest eigenvalues, then $X=V\sqrt{\Lambda }$, *X* is an *N* × *r* matrix with the coordinates *x*_*i*_ as its rows. Thus the coordinates in an r-dimensional can be recovered from the distance matrix *D*. In coevolution analysis, the distance matrix *D* derived from the correlation coefficients of pairwise proteins is projected onto two dimensions (r = 2) by MDS for visualizing the relative interactions of proteins.

## 3 Results

### 3.1 Phylogenetic analysis of Ebola virus by Fourier transform

**Fig 2:**
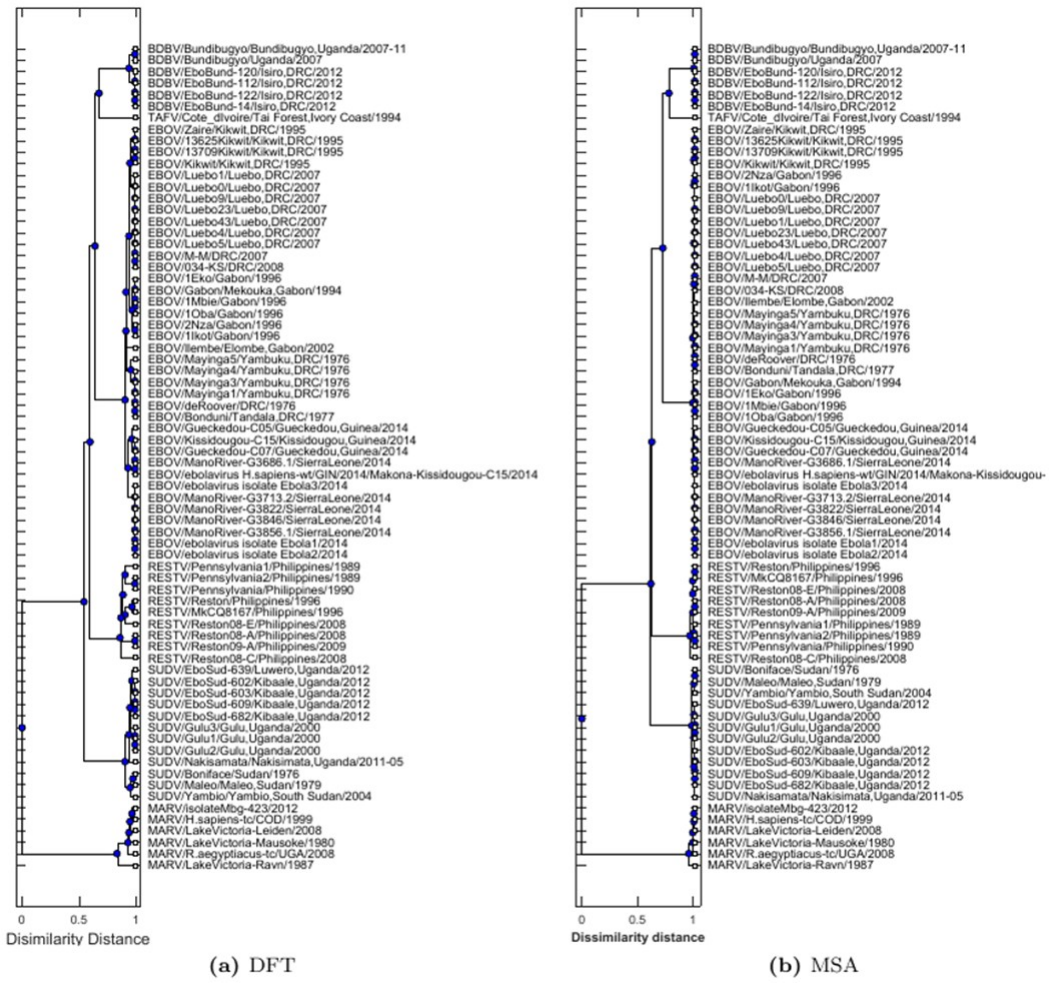
Phylogenetic analysis of Ebola virus. (a) Phylogenetic tree constructed by the DFT distance of NP sequences of Ebola virus. (b) Phylogenetic tree constructed by MSA of NP sequences of Ebola virus.

To demonstrate the effectiveness of the DFT measure of protein sequences, we apply the phylogenetic analysis of Ebola virus using NP protein by the DFT method and multiple sequence alignment (MSA). The result shows that the phylogenetic trees from the DFT method contain rich hierarchical information about different virus species (Fig 2(a)). The DFT method can thus identify small difference among closely related species (Fig 2(a)). However, the phylogenetic tree from the MSA method cannot show the difference of closely related species (Fig 2(b)), thus these species cannot be well separated using the MSA method. This result demonstrates that a phylogenetic tree from MSA may not reflect the true physiochemical changes of amino acid in protein mutations, and may cause high false positive rate in coevolution analysis. The similar results are observed in the phylogenetic analysis of other 6 proteins in Ebola virus in supplementary materials (S3 File). These results suggest that our DFT method holds promises in differentiating mutations and outperforms the sequence alignment methods in phylogenetic analysis.

### 3.2 Coevolution analysis of protein-protein interactions within Ebola virus

To assess our proposed DFT method in detecting PPIs, we use the DFT method to investigate the interactions of all seven proteins in Ebola virus. The Pearson correlation of the distance matrices of seven proteins in coevolution analysis by the DFT method is listed in Table 2. High correlation between the two proteins indicates an interaction between these two proteins. For example, the correlation coefficient of VP24 and VP40 is 0.9745 (Table 2), indicating that these two matrix proteins interact. This is in agreement with experimental studies [27].

**Table 2:** Pearson correlation coefficient of proteins in Ebola genomes by DFT method.

	GP	NP	VP24	VP30	VP35	VP40	L
GP	1.0000	0.7745	0.7238	0.7843	0.7165	0.7967	0.7900
NP		1.0000	0.9883	0.9419	0.6698	0.9873	0.8064
VP24			1.0000	0.9289	0.5761	0.9745	0.7757
VP30				1.0000	0.5849	0.9449	0.7720
VP35					1.0000	0.6348	0.7584
VP40						1.0000	0.7892
L							1.0000

After the correlation matrix for pair-wise proteins is obtained from the DFT method (Table 1), relative distances of PPIs among of seven proteins in Ebola virus are illustrated using MDS in Fig 2. From the coevolution analysis, we can see there are strong coevolution relationship between the following protein pairs (NP-VP24, NP-VP30, NP-VP40, VP24-VP30, VP24-VP40, and VP30-VP40). This result agrees with previous experimental studies that showed NP, VP30, and L, form the nucleocapsid complex (NC) for genome transcription [11]. Furthermore, previous study indicated that without the viral transcription activator VP30, three proteins NP, VP35, and L are sufficient to mediate viral replication in a reconstituted replication and transcription system [28].

NP protein plays a central role in protein interaction network. The proteins NP, VP24, VP30 and VP40 have a close relationship. Previous experimental study suggested that VP40 appears to physically interact with NP by detection of NP in VP40-containing VLPs [29]. The coefficient of VP40 and NP protein is 0.9873. This result confirms the interaction between NP and VP40 protein.

It shall be noted that from co-immunoprecipitation study, NP protein interacts with both VP24 and VP35, the four proteins (NP, VP24, VP35, and VP40) are necessary and sufficient to mediate assembly of an NC with structure [30], but our coevolution study indicates a weak interaction between NP and VP35. This inconsistent observations need future more studies.

**Fig 3:**
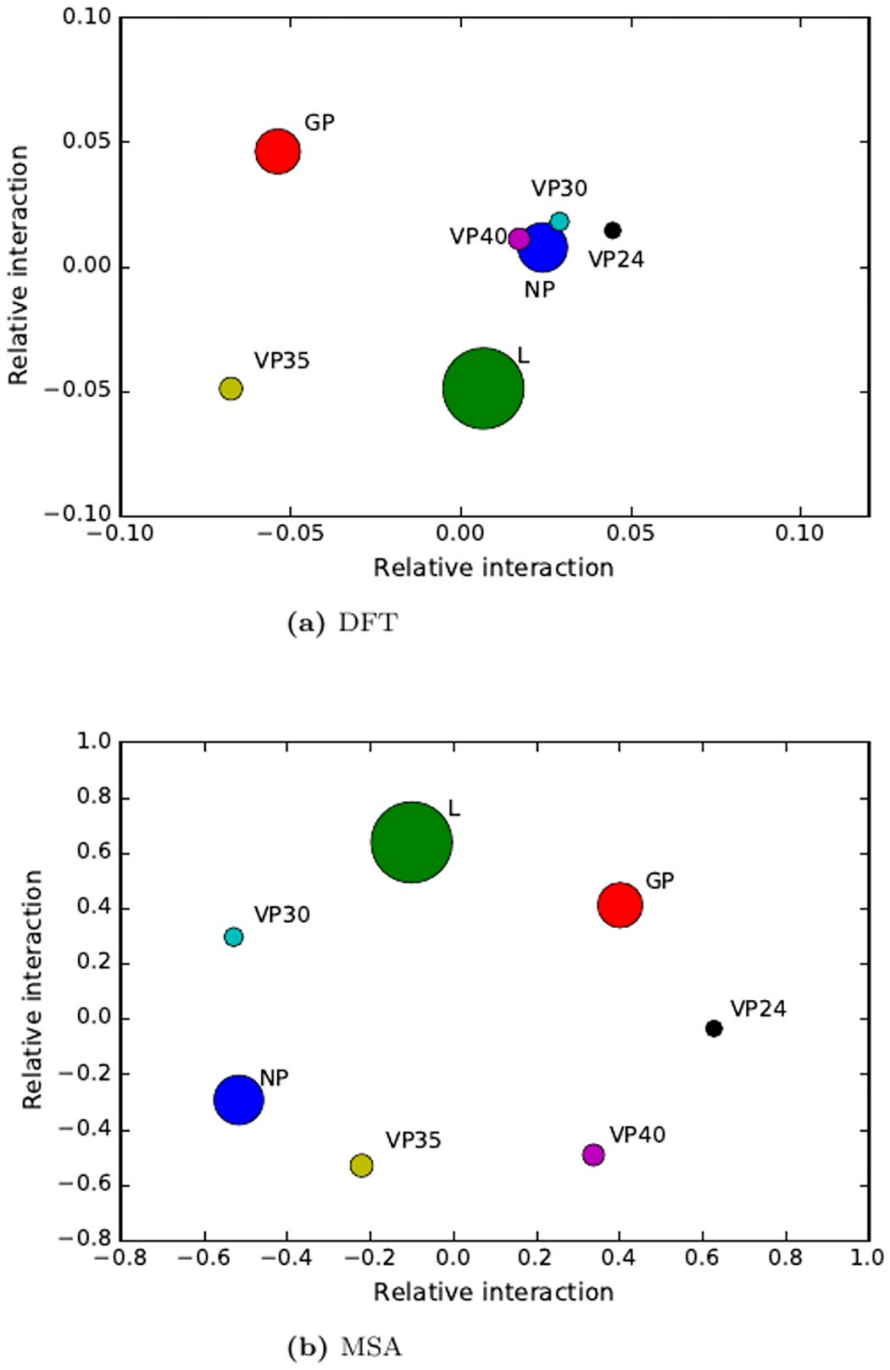
Multidimensional scaling analysis of PPIs in Ebola virus by coevolution. (a) DFT method. (b) MSA method in MirrorTree.

The VP24 protein of Ebola virus is a secondary matrix protein and minor component of virions. VP24 is critical matrix protein in Ebola virus [31]. Previous experiments show that protein VP24 inhibits transcription and replication of the EBOV genome, indicating VP24 plays a regulatory role in virus replication [31]. VP24 interacts with NP, VP30 and VP40 from this coevolution study (Table 2 and Fig 3(a)). Our coevolution analysis shows there is strong interaction between VP24 and NP, we infer that one possible molecular mechanism that VP24 protein reduces transcription is through interacting with NP in the nucleocapsid complex. This molecular mechanism is confirmed by previous experimental study [31]. In addition, an immunoprecipitation study indicates there is no interaction between VP24 and VP35 [31], our coevolution study also validates that there is no strong interaction between VP24 and VP35.

The membrane-associated matrix protein VP40 of Ebola virus is the most abundant virion protein and plays a key role in virus assembly and budding in the form of virus-like particles (VLPs) [27]. Our coevolution analysis shows that VP40 protein interacts with NP, VP24, and VP30. Previous study suggested that combinations of GP/VP24/NP expression can enhance release of VP40 VLPs [29]. The GP and NP mediated enhancement of release VP40 VLPs may be due to interactions with VP40 [29]. However, there has been no report on interaction of VP24 and VP40 in previous research. Our coevolution study indicates that VP24 also interacts with VP40 to facilitate release of VP40 VLPs.

Ebola virus VP30 is an essential transcription activator, and RNA binding protein of viral transcription. In viral particles, VP30 is closely associated with the nucleocapsid complex, but how VP30 activates transcription is still unclear. Phosphorylation of VP30 regulates viral transcription and replication by modulating interaction with the nucleocapsid proteins VP35 and NP [32]. Our coevolution analysis confirms that VP30 interacts with NP inside the nucleocapsid complex. In addition, our coevolution study result indicates the interaction of VP30 with two matrix proteins VP24 and VP40, which has not been reported before. This prediction needs future experimental confirmation. Furthermore, previous studies show that both VP40 and VP24 inhibits transcription and replication of the EBOV Genome [31, 33, 34]. However, no studies have been performed investigating the mechanism of transcription regulation by the matrix proteins. From the coevolution analysis, we may infer that a possible mechanism of the inhibition of VP24 and VP40 on transcription is to interact with transcription factor VP30.

Our coevolution analysis indicates VP35 does not have strong interactions with other proteins. VP35 acts as RNA polymerase cofactor in the transcription and replication complex and plays an essential role in viral RNA synthesis and interacts with the viral nucleoprotein [35]. It is also an inhibitor of the type I IFN response in Ebola virus-infected cells and may be an important determinant of Ebola virus virulence [36]. Three proteins NP, VP35, and L, are sufficient to mediate replication and transcription of virus [37], but transcription requires needs VP30 as activation factor [28]. From our coevolution study, we can postulate that interaction between NP and VP30 is important in the activation of replication and transcription because there is little interaction between VP35/L and VP30.

It is noted that the Ebola RNA polymerase (L) does not have strong coevolution with other proteins. It is reasonable because RNA polymerase works as making reliable copies of virus genomes. Dramatic mutation in RNA polymerase may produce instable virus genomes. Another possible reason for the loose coevolution between RNA polymerase and other 6 proteins is that a virus with high mutation in RNA polymerase may not survive.

The glycoprotein (GP) is the only viral protein on the surface and is therefore responsible for receptor binding and membrane fusion, mediating attachment and entry of the virus into host cells [38]. The coevolution analysis indicates this protein has weak interaction with other proteins, the reason is that this protein is on viral surface.

We compare our DFT method with the state-of-art MirrorTree method in PPI prediction [2, 25]. The correlation of coevolution of the seven proteins in Ebola virus by the MirrorTree is listed in Table 3. The results in Table 3 shows that the almost all correlations from the MirrorTree method are larger than 0.95, indicating all these seven proteins highly interact. Thus the result from MirrorTree might contain many false positives. The relative interaction of these 7 proteins projected by MDS shows dispersed pattern (Fig 3(b), indicating the interactions among the proteins are not clear. Comparison of the correlations from the DFT method and the MirrorTree (Tables 2 and 3, Fig 3(a) and 3(b)) demonstrates that the DFT method has lower false positive than the MirrorTree method. The DFT method can truly capture the physiological impact of different amino acid mutations, but MSA based MirrorTree considers the same impact of different amino acid mutations.

**Table 3:** Pearson correlation of proteins in Ebola genomes by MirrorTree.

	GP	NP	VP24	VP30	VP35	VP40	L
GP	1.0000	0.974	0.939	0.965	0.979	0.914	0.980
NP		1.0000	0.975	0.987	0.696	0.963	0.988
VP24			1.0000	0.981	0.969	0.979	0.972
VP30				1.0000	0.983	0.968	0.983
VP35					1.0000	0.953	0.987
VP40						1.0000	0.954
L							1.0000

### 3.3 Coevolution analysis of protein-protein interactions in influenza A virus

Influenza A viruses belong to the Orthomyxoviridae family of negative-sense, single-stranded RNA viruses. The virus genome is composed of 8 segments, encoding for 11 proteins: HA (hemagglutinin), NA (neuraminidase), NP (nucleoprotein), M1 (matrix protein), M2, NS1 (Non-structural protein 1), NEP (nuclear export protein), PA (Polymerase acidic protein), PA-X, PB1 (polymerase basic 1), and PB2 [39, 40]. HA and NA are the two envelope glycoproteins on the surface of influenza virions and play critical roles in influenza infection. NS1 is a multifunctional protein and a virulence factor and NS2 involves in nuclear export of viral ribonucleoprotein complexes [41]. An interaction between the cytoplasmic tail of M2 and M1 promotes the recruitment of the internal viral proteins and RNA to the plasma membrane for efficient virus assembly [42]. NP encapsidates the RNA polymerase complex (PB1, PB2 and PA) and the 8 segment to form the viral ribonucleoproteins (vRNPs) [43]. The viral particle contains eight vRNPs, the surface glycoproteins HA and NA, the matrix proteins (M1 and M2) and the NEP protein. NS1 protein is not incorporated in the virus. It interacts with a variety of cellular components in the cytoplasm and nucleus. NEP (formerly known as NS2) protein mediates the export of vRNPs from the nucleus to the cytoplasm and associates with the matrix M1 protein [44]. In addition, resulting from a frameshift [45], the PA gene encodes a second small protein, called PA-X, which hijacks the host immune response through host protein shutdown mechanisms, thereby modulating the antiviral pathways [46, 47].

**Fig 4:**
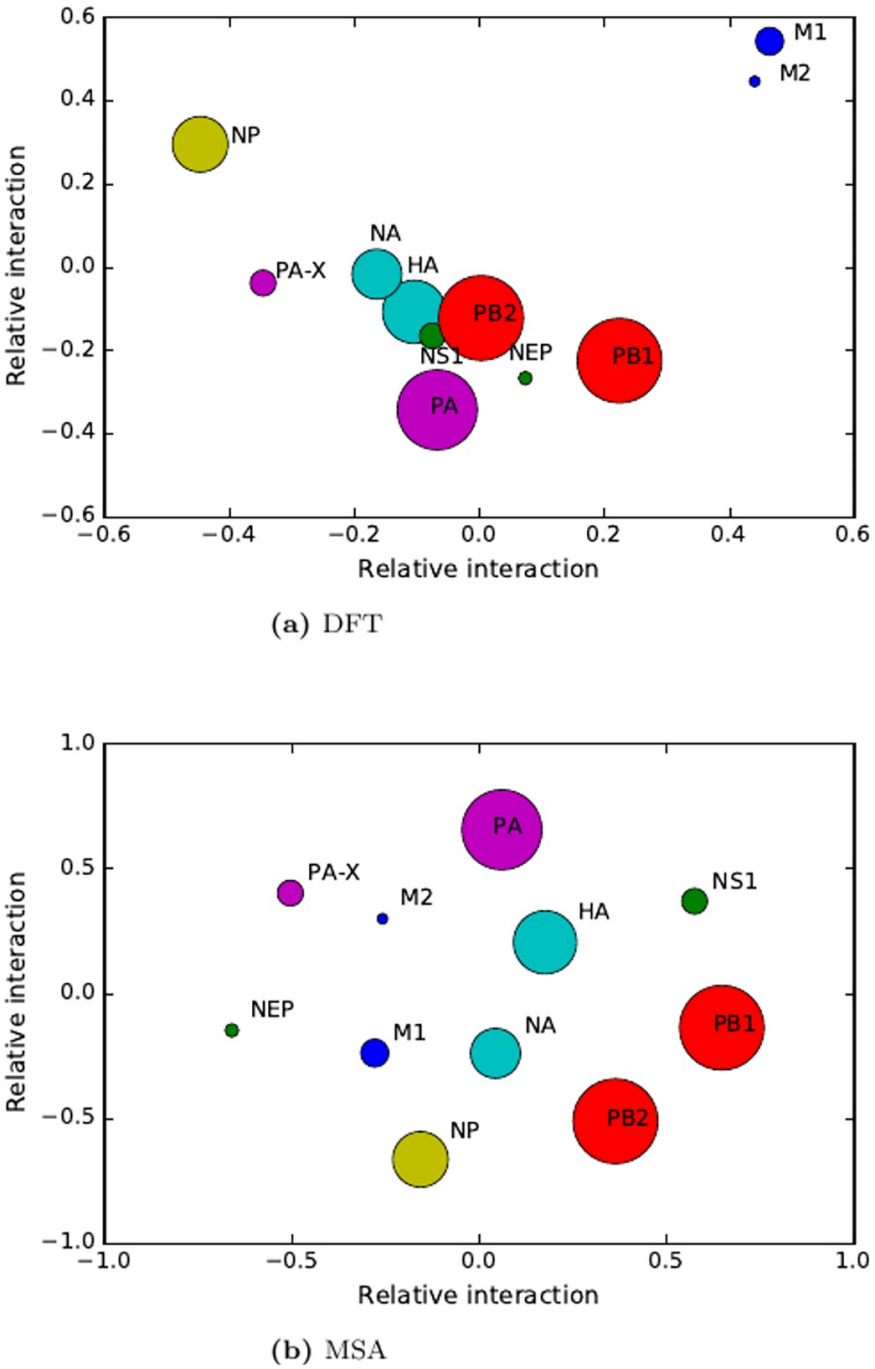
Multidimensional scaling analysis of PPIs in influenza A virus by coevolution. (a) DFT method. (b) MSA method in MirrorTree.

We assess the DFT coevolution method in identifying the protein-protein interactions among these 11 proteins in influenza A virus. The coevolution analysis is performed on the 60 influenza virus genomes. The GenBank access numbers of the influenza virus genomes are listed in supplementary materials (S2 File). The Pearson correlations of the protein-protein interactions is shown in Table 4. The relative interaction distances derived from the Pearson correlations are shown in Fig 4(a). From the coevolution analysis, we can see there are strong correlations the following protein pairs and groups (HA-NA, HA-NS1, HA-PB2, M1-M2, NS1-PB2, NS1-PA, NA-PA-X, PA-PB1-PB2, NA-vRNPs, HA-vRNPs, NS1-vRNPs, NEP-vRNPs).

**Table 4:** Distance correlation of proteins in influenza A genomes by the DFT method.

	HA	M1	M2	NA	NP	NEP	NS1	PA	PA-X	PB1	PB2
HA	1	0.1548	0.2653	0.8375	0.4276	0.7504	0.9454	0.7713	0.8056	0.6249	0.8651
M1		1	0.9507	0.1119	0.0998	0.0970	0.1119	0.0588	-0.0420	0.1540	0.1931
M2			1	0.1818	0.0777	0.1746	0.2451	0.1771	0.1174	0.2827	0.7660
NA				1	0.3783	0.6340	0.7911	0.6091	0.7865	0.5359	0.7693
NP					1	0.3187	0.3889	0.2689	0.6366	0.3142	0.3983
NEP						1	0.7647	0.7333	0.6690	0.8274	0.7307
NS1							1	0.8126	0.6295	0.6832	0.8434
PA								1	0.5457	0.6687	0.7132
PA-X									1	0.4023	0.5554
PB1										1	0.6892
PB2											1

We verify these interactions by curating literatures of experimental studies. In virus, HA attaches to sialic acid on host cell surface to initiate virus infection [48]; NA removes by cleavage of sialic acid from cell receptor which HA binds to facilitate virus release from infected cells by cleavage of sialic acids between the host cell and the HA protein [49, 50]. The interaction between HA and NA is well studied by genetic method [51] and protein structures [52]. Both HA and PB2 are critical for virus virulence [53, 54]. HA mutations may enhance replication and virulence [55]. These studies indicate HA and PB2 interact with other. The matrix protein (M1) is the major structural protein, and underlies the viral envelope and the M2 protein has a proton-selective ion channel activity. A direct interaction between M1 and the M2 cytoplasmic tail has been identified by genetic mutations and biochemical studies [42]. NS1 and NEP interaction is identified by yeast two-hybrid system [41]. NS1 protein interacts with the PB2 in vRNP by *in vivo* genetic study [56]. NS1 protein also interacts with PA evidenced by immunoprecipitation experiments [56]. Since NEP is nuclear export protein for transporting polymerase complex [57, 58], NEP has close interaction with the polymerase complex (PB1, PB2 and PA) as shown in Fig 4(a). PA-X is a newly found protein with a function to trigger host RNA degradation [46, 47]. The associated proteins with PA-X have not been identified in previous studies. The coevolution analysis suggests that PA-X is associated with NA or NP (Fig 4(a)). In summary, the protein interactions from the coevolution DFT analysis have also been identified by experimental studies. We compare the proposed coevolution DFT method with the MSA based MirrorTree method for identifying PPIs in influenza A virus. The Pearson correlation of coevolution and relative interactions of the 10 proteins in influenza virus by the MirrorTree are shown in Table 5, and Fig 4(b), respectively. The result in Table 5 shows that the major correlations from the MirrorTree method are large. The relative interactions of the proteins projected by MDS are widely scattered (Fig 4(b)). It is difficult to identify positive interactions from these high correlations by the MirrorTree method. However, the correlations in DFT method are in reasonable ranges, and can distinguish the positive interactions from the weak or non-interacted protein pairs. This comparison demonstrates that the proposed DFT method outperforms the MirrorTree method for PPIs prediction.

**Table 5:** Distance correlation of proteins in influenza A genomes by the MirrorTree method.

	HA	M1	M2	NA	NP	NEP	NS1	PA	PA-X	PB1	PB2
HA	1.000	0.650	0.650	0.996	0.808	0.841	0.878	0.788	0.987	0.819	0.756
M1		1.000	0.903	0.635	0.855	0.847	0.872	0.866	0.763	0.902	0.902
M2			1.000	0.631	0.845	0.849	0.848	0.830	0.293	0.884	0.832
NA				1.000	0.796	0.828	0.866	0.783	0.988	0.807	0.807
NP					1.000	0.960	0.971	0.965	0.968	0.985	0.931
NEP						1.000	0.970	0.951	0.968	0.969	0.916
NS1							1.000	0.957	0.974	0.982	0.933
PA								1.000	0.944	0.972	0.965
PA-X									1.000	0.968	0.968
PB1										1.000	0.956
PB2											1.000

### 3.4 Coevolution analysis of protein-protein interactions in *E.coli*

To test proposed DFT method on coevolution analysis in large scale, we evaluate the relationship of Pearson correlation by the DFT method and the genetic interaction score (GI) of protein pairs in *E.coli*. Examining the phenotypes resulting from pairs of mutations may offer a way to understand how these genes intersects [59]. Genetic interactions are classified as either positive or negative. When a loss of function mutation of two given genes results in exceeding the fitness that is from individual effects of deleterious mutations, then GI score is positive; when it decreases the fitness, the GI score is negative.

**Fig 5:**
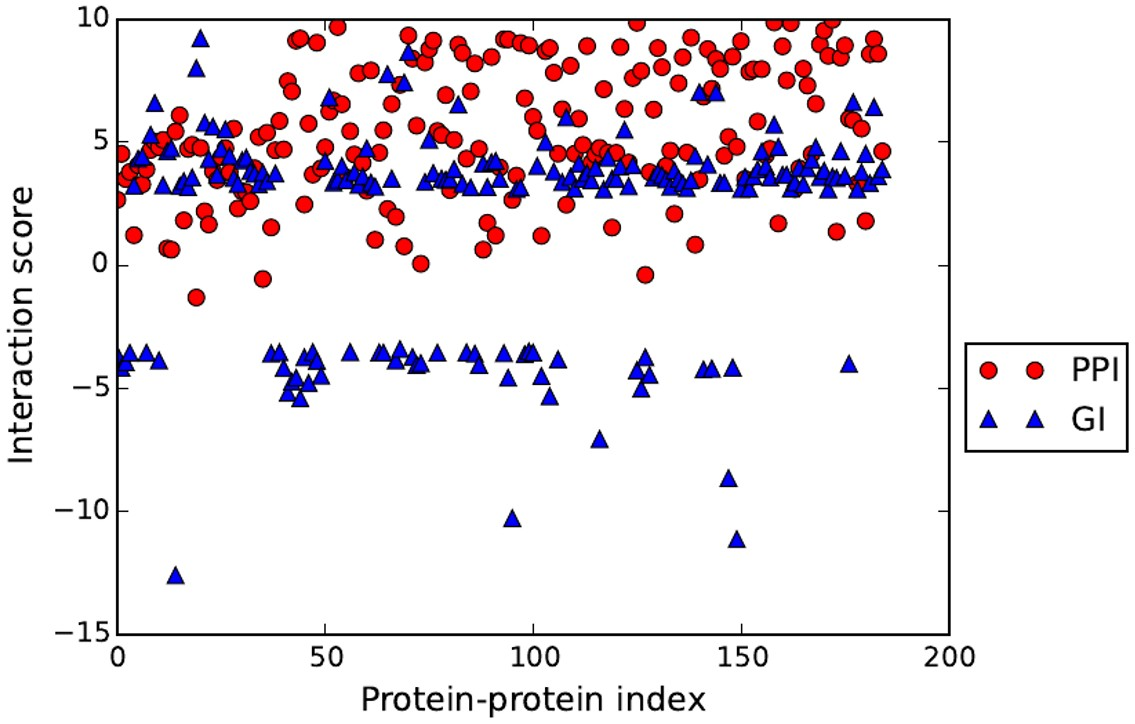
Relationship between PPIs by the coevolution DFT method and GIs of protein pairs in *E.coli*. The PPI scores are represented by the Pearson correlation and scaled by 10.

Genetic interactions of two genes may reflect the protein-protein interactions. It is expected that there is a correlation between GI and PPI [60]. For a pair of proteins, if the GI score is positive, the PPI is expected since the interacted proteins may occur within a pathway. If the GI score is zero or negative, the PPI is not expected or weak since the two proteins could be in different pathways. Here we examine this perspective relationship between PPIs and GIs using protein pairs of genetic interaction in *E.coli* [61]. The GenBank access numbers of the *E.coli* genomes are listed in supplementary materials (S2 File). The GI data set with GI scores from *E.coli* is from [61]. The result of 185 PPI pairs shows that there is correlated relationship between PPI and GI scores for the tested protein pairs (Fig 5). If the threshold value of correlation coefficient of PPIs is set as 0.2, the DFT method may identify 88.10% positive GI protein pairs. The accuracy for the true GI positives in predicated PPIs is 63.24%. Considering that relationship of PPIs and GIs is nonlinear, this result may indicate that the proposed coevolution DFT method may identify PPIs that are correlated to GIs.

It is noted that experimental studies on protein-protein interactions may pose many challenges, especially the PPIs from experiments may contain high false positives and false negatives. There are several large-scale studies on protein-protein interaction network in *E.coli* [59, 61–67], however, due to the complexity of PPIs, the PPIs identified in *E.coli* are incomplete and sometimes inconsistent. Coevolution analysis for PPIs provides complementary Information for reducing the false positives and negatives and ensures comprehensive coverage of an interactome.

## 4 Discussions

The rational of the algorithm is based on the fact that hydrophobical properties of amino acids are the primary force in determining protein structures. Most protein molecules have a hydrophobic core, which is not accessible to solvent and a polar surface in contact with the environment although membrane proteins follow a different pattern. While hydrophobic amino acid residues build up the core, polar and charged amino acids preferentially cover the surface of the molecule and are in contact with solvent due to their ability to form hydrogen bonds. Very often they also interact with each other: positively and negatively charged amino acids form so called salt bridges, while polar amino acid side chains may form side chain-side chain or side chains-main chain hydrogen bonds. It has been observed that all polar groups capable of forming hydrogen bonds in proteins do form such bonds. Since these interactions are often crucial for the stabilization of the protein three-dimensional structure, they are normally conserved.

Although the DFT coevolution method computes the interaction score of two proteins based on the similarity of their phylogenetic trees, the method does not require the construction of phylogenetic trees but analyzes the underlying distance matrices, which makes this approach independent of tree construction methods.

With the advancement of next generation sequencing methodologies, a large collection of gene and protein sequences across different species are available. These sequences contain rich coevolution information and enable us to identify PPIs using computational approaches. We can better understand the protein functional network and make substantial advances in functional genomics [68, 69]. Furthermore, the computational method can increase confidence levels for PPI identification by experimental studies. In addition, protein interaction analysis is increasingly important in rapidly characterizing pathogens of infectious disease such as Ebola virus and MERS virus. Identifying and understanding PPIs in virus genomes may shed lights on the virus replication and infection. The results of PPIs in virus genomes may find drug targets and is useful in developing antivirus vaccines.

Our study on the PPIs in Ebola virus and influenza virus clearly demonstrates the effectiveness of our method in identification of PPIs. The core component of our method in coevolution analysis for detecting PPIs is the precise measurement for protein sequence mutations. The precise measurement method may also have significant applications in precise medicine. In precise medicine, substitution mutations in protein sequences are caused by SNPs (single nucleotide polymorphism), which are genetic contributions to complex diseases, such as cancer [15]. Most of the SNP analysis methods only consider the type of SNPs and positions, but our method can compare and characterize SNPs in context of full sequences and chemical properties of amino acids. Thus our method for comparing protein sequences can reveal and predict true impacts of SNPs on important diseases such as cancer and Parkinson disease. In general purpose, the our method is useful in not only theoretical perspective, but also in directing pharmaceutical development, and precise medicine.

## 5 Conclusions

In this study, we develop and extend a quantitative method that identifies interacting proteins by coevolution analysis. Our method employs DFT analysis of chemical properties of amino acids in position context of protein-protein interactions. The method offers evidences of coevolution for protein interactions in Ebola and influenza viruses. Most of the interactions identified by this coevolution study are in agreement with previous studies. We also find that there is strong coevolution between two matrix proteins VP24 and VP40, between matrix proteins VP24/VP40 and transcription factor VP30.

## Supporting information

S1 File Fourier transform of VP24 protein of Ebola virus. (PDF)

S2 File GenBank access numbers of genes or genomes of Ebola virus, influenza virus and *E.coli*. (XLSX)

S3 File Phylogenetic analysis of proteins in Ebola virus. (PDF)
