# Supplementary material for: A coevolution analysis for identifying protein-protein interactions by Fourier transform
Source: PLoS One. 2017 Apr 21;12(4):e0174862. doi: 10.1371/journal.pone.0174862 (PMC5400233; doi:10.1371/journal.pone.0174862)
Supplement: S1 File — (PDF) [file pone.0174862.s001.pdf]

A Coevolution Analysis for Identifying Protein-Protein Interactions by Fourier Transform

Changchuan Yin[1], Stephen S.-T. Yau[2*]

1 Department of Mathematics, Statistics and Computer Science,The University of Illinois at Chicago, Chicago, IL 60607-7045, USA
2 Department of Mathematical Sciences, Tsinghua University, Beijing 100084, China

Fourier transform of VP24 protein in Ebola virus

The following program is to compute Fourier transform of VP proteins sequence (1-50 amino acid positions) in Ebola virus.

Python programs

```
import numpy as np
from scipy.fftpack import fft

def DFTHydrophobicity(seq):
  AAHydrophobicity = {'A': 1.8, 'R': -4.5, 'N': -3.5,'D': -3.5,\
  'C':2.5, 'Q': -3.5,'E':-3.5,'G':-0.4,'H': -3.2,'I':4.5,'L':3.8,\
  'K': -3.9,'M': 1.9,'F':2.8,'P':-0.9,'S': -0.8,'T':-0.7,'W':-0.9,\
  'Y':-1.3,'V':4.2,'U':0.0,'X':0.0} #X is for padding zeros
  n=len(seq)
  listAA=np.zeros(n) #define zeros list of length n
  print('1.Symbolic protein sequence:\n',seq)

  for i in range(0,n):  # use index from 0 to n
    aa=seq[i]
    listAA[i]=AAHydrophobicity[aa]
  print('2.Numerical sequence:\n',listAA)

  fftAA = fft(listAA)

  ps=np.abs(fftAA[0:n])
  ps=pow(ps,2)


  R=fftAA.real
  I=fftAA.imag

  vRI=[]

  for i in range (0,n):
```

```
        vRI.append(R[i])
    for i in range(0,n):
        vRI.append(I[i])

    print('3.DFT of the numerical sequence:\n',fftAA)

    return [ps,vRI]

# Test: first 50 amino acids of VP24 protein
#>AIE11921.1 VP24 [Zaire ebolavirus]
seq='MAKATGRYNLISPKKDLEKGVVLSDLCNFLVSQTIQGWKVYWAGIEFDVT\
HKGMALLHRLKTNDFAPAWSMTRNLFPHLFQNPNSTIESPLWALRVILAA\
GIQDQLIDQSLIEPLAGALGLISDWLLTTNTNHFNMRTQRVKEQLSLKML\
SLIRSNILKFINKLDALHVVNYNGLLSSIEIGTQNHTIIITRTNMGFLVE\
LQEPDKSAMNRKKPGPAKFSLLHESTLKAFTQGSSTRMQSLILEFNSSLAI'
seq=seq[0:50]
[ps,vRI]=DFTHydrophobicity(seq)
```

Result of the Python programs

The result of the program is as follows, showing the numerical representation of the protein sequence and its Fourier transform coefficients.

```
1.Symbolic protein sequence:
 MAKATGRYNLISPKKDLEKGVVLSDLCNFLVSQTIQGWKVYWAGIEFDVT
2.Numerical sequence:
 [ 1.9   1.8  -3.9   1.8  -0.7  -0.4  -4.5  -1.3  -3.5   3.8
   4.5  -0.8  -0.9  -3.9  -3.9  -3.5   3.8  -3.5  -3.9  -0.4
   4.2   4.2   3.8  -0.8  -3.5   3.8   2.5  -3.5   2.8   3.8
  4.2  -0.8  -3.5  -0.7   4.5  -3.5  -0.4  -0.9  -3.9   4.2
  -1.3  -0.9   1.8  -0.4   4.5  -3.5   2.8  -3.5   4.2  -0.7]
3.DFT of the numerical sequence:
 [   2.00000000  +0.j           -10.05609249+12.07554328j
  23.10414000  +4.85505075j  -12.08537106  +4.92520703j
  -3.80405050+12.79259628j   30.95951949  -3.62413697j
  -3.67895877-15.28872799j    7.88604472  -4.62125878j
 -24.95072022  +5.27475602j    8.35277509+23.28276524j
  31.53773076  +7.8823957j   -30.41582296  +5.25522425j
  22.40105719-10.03739826j    -7.01295130  -0.13009415j
   0.11893514-11.68915139j    9.94048051+16.04028152j
  14.02191734-11.18154017j  -22.87419050-15.67641379j
  13.75713104-16.46518361j   -2.22513158+14.23051355j
 -16.53773076  -4.46013878j   -9.15040650-16.50879912j
  16.51259726-37.7952284j      2.33114658-22.78838915j
  -2.23204849  -9.10374548j   21.20000000  +0.j  -2.23204849  +9.10374548j
   2.33114658+22.78838915j   16.51259726+37.7952284j
  -9.15040650+16.50879912j  -16.53773076  +4.46013878j
  -2.22513158-14.23051355j   13.75713104+16.46518361j
 -22.87419050+15.67641379j   14.02191734+11.18154017j
   9.94048051-16.04028152j    0.11893514+11.68915139j
  -7.01295130  +0.13009415j   22.40105719+10.03739826j
 -30.41582296  -5.25522425j   31.53773076  -7.8823957j
   8.35277509-23.28276524j  -24.95072022  -5.27475602j
   7.88604472  +4.62125878j   -3.67895877+15.28872799j
```

```
 30.95951949  +3.62413697j    -3.80405050-12.79259628j
-12.08537106  -4.92520703j    23.10414000  -4.85505075j
-10.05609249-12.07554328j]
```
