# Supplementary material for: A coevolution analysis for identifying protein-protein interactions by Fourier transform
Source: PLoS One. 2017 Apr 21;12(4):e0174862. doi: 10.1371/journal.pone.0174862 (PMC5400233; doi:10.1371/journal.pone.0174862)
Supplement: S3 File — (PDF) [file pone.0174862.s003.pdf]

A Coevolution Analysis for Identifying Protein-Protein Interactions by Fourier Transform

Changchuan Yin[1], Stephen S.-T. Yau[2*]

1 Department of Mathematics, Statistics and Computer Science,The University of Illinois at Chicago, Chicago, IL 60607-7045, USA
2 Department of Mathematical Sciences, Tsinghua University, Beijing 100084, China

Phylogenetic analysis of proteins in Ebola virus by Fourier transform and multiple sequence alignment (MSA)

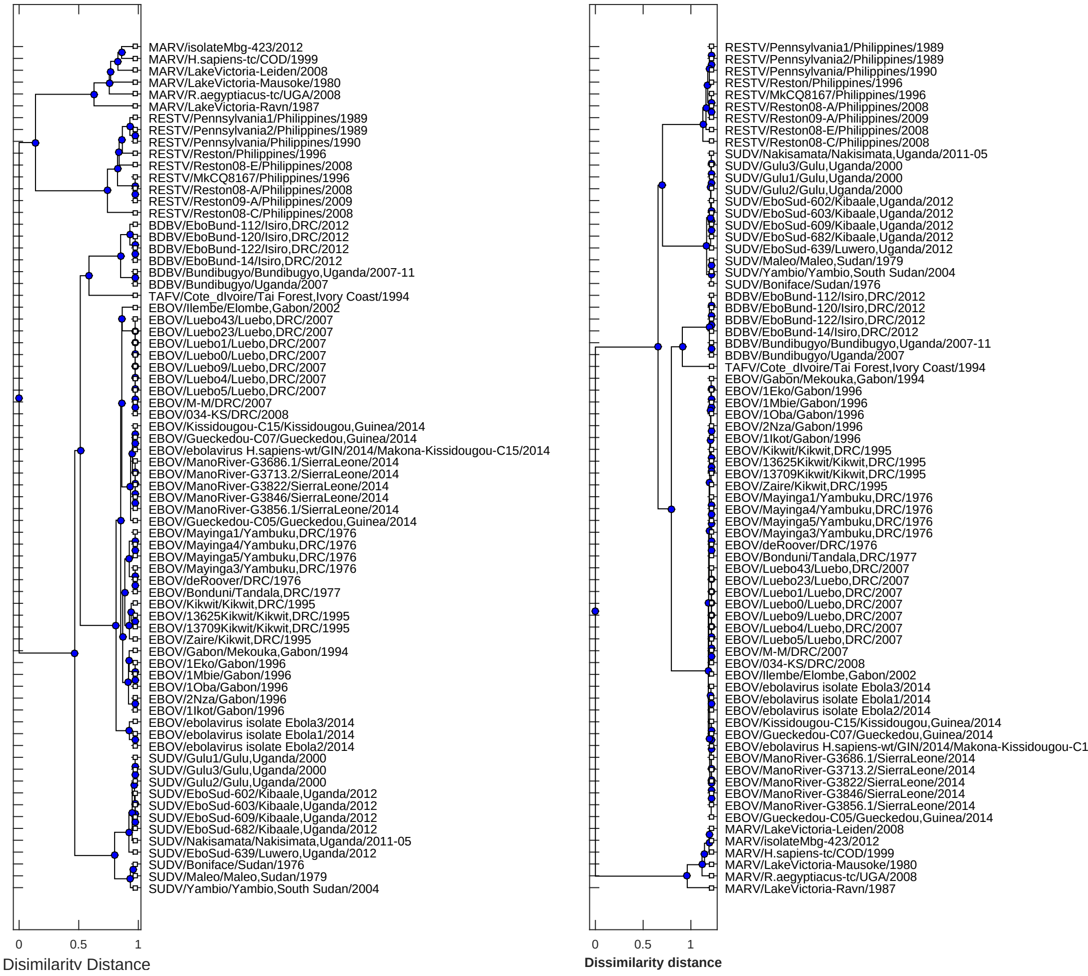

(a) DFT **(b)** MSA

Figure 1. Phylogenetic analysis of Ebola virus. (a) Phylogenetic tree constructed by the DFT distance of GP sequences of Ebola virus. (b) Phylogenetic tree constructed by MSA of GP sequences of Ebola virus.

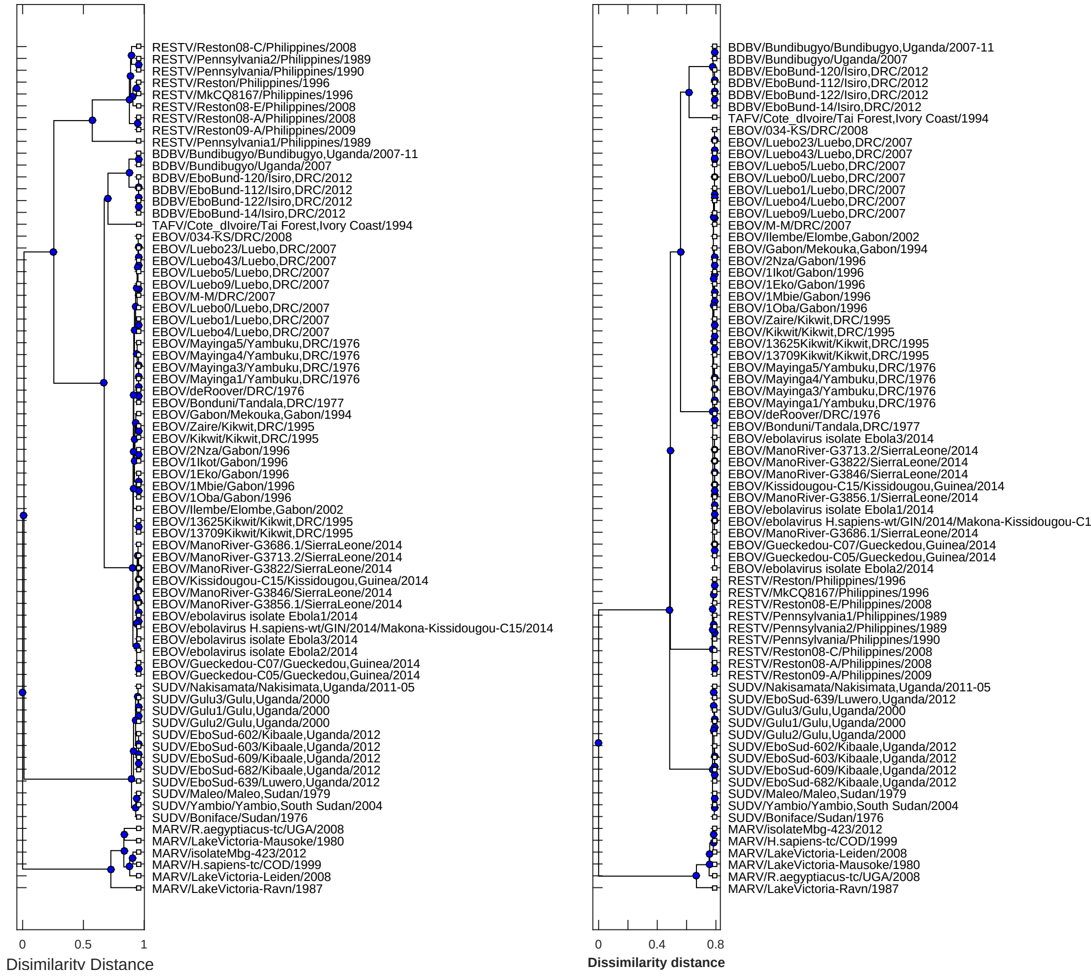

(a) DFT **(b)** MSA

Figure 2. Phylogenetic analysis of Ebola virus. (a) Phylogenetic tree constructed by the DFT distance of L sequences of Ebola virus. (b) Phylogenetic tree constructed by MSA of L sequences of Ebola virus.

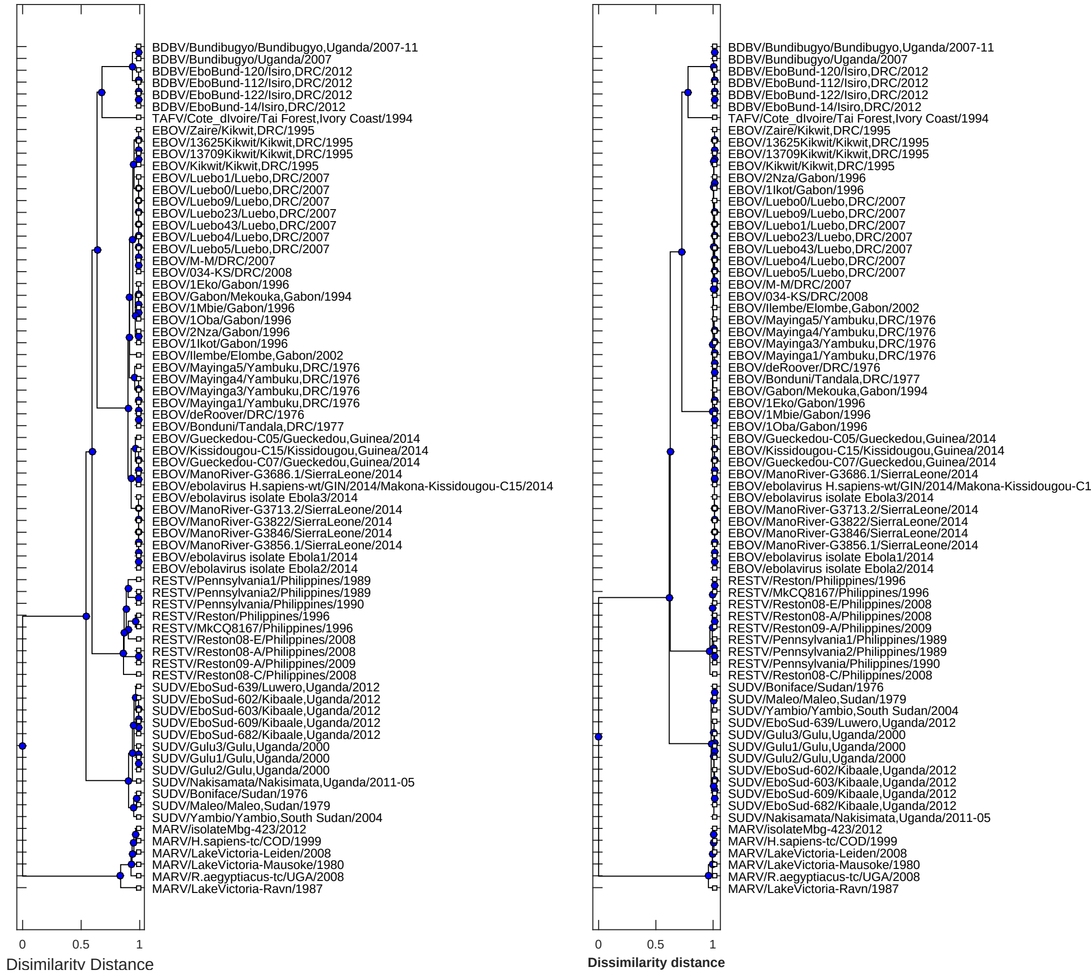

(a) DFT **(b)** MSA

Figure 3. Phylogenetic analysis of Ebola virus. (a) Phylogenetic tree constructed by the DFT distance of NP sequences of Ebola virus. (b) Phylogenetic tree constructed by MSA of NP sequences of Ebola virus.

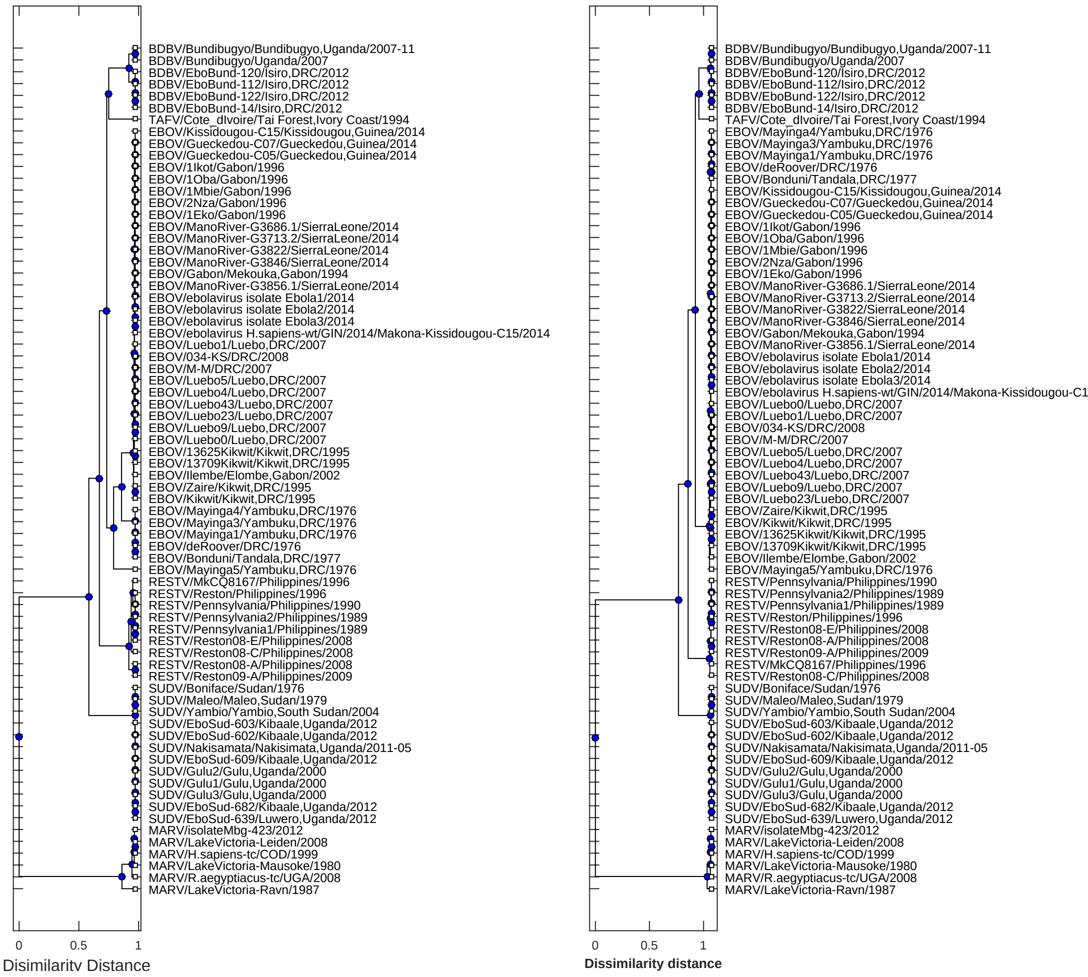

(a) DFT **(b)** MSA

Figure 4. Phylogenetic analysis of Ebola virus. (a) Phylogenetic tree constructed by the DFT distance of VP24 sequences of Ebola virus. (b) Phylogenetic tree constructed by MSA of VP24 sequences of Ebola virus.

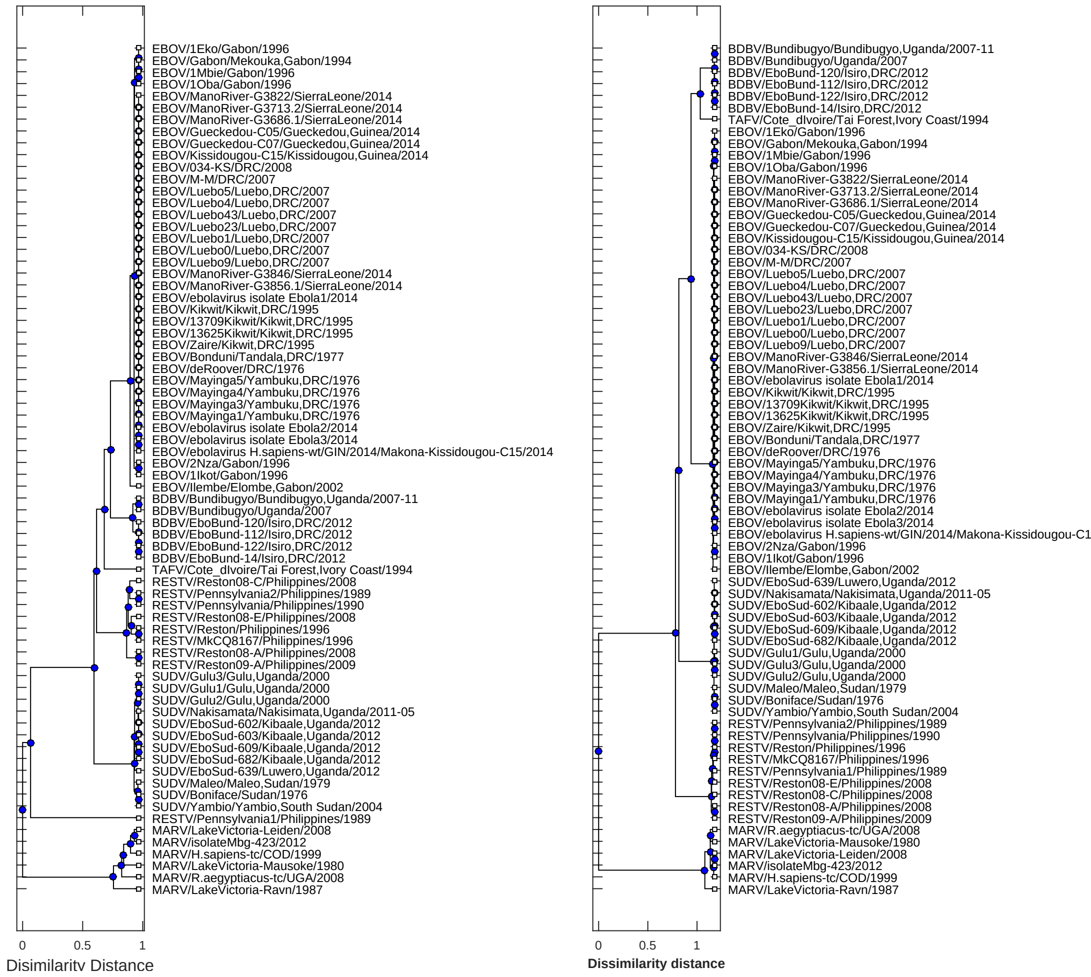

(a) DFT **(b)** MSA

Figure 5. Phylogenetic analysis of Ebola virus. (a) Phylogenetic tree constructed by the DFT distance of VP30 sequences of Ebola virus. (b) Phylogenetic tree constructed by MSA of VP30 sequences of Ebola virus.

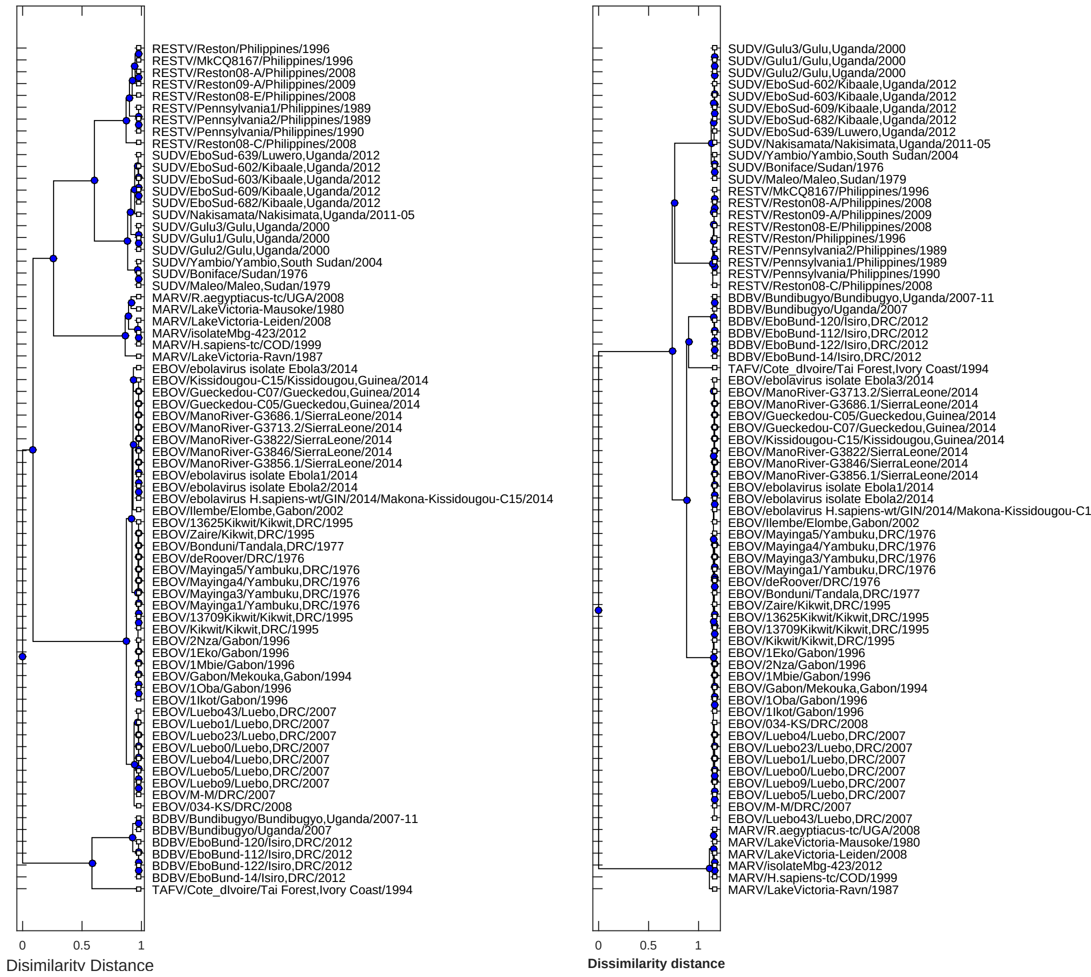

(a) DFT **(b)** MSA

Figure 6. Phylogenetic analysis of Ebola virus. (a) Phylogenetic tree constructed by the DFT distance of VP35 sequences of Ebola virus. (b) Phylogenetic tree constructed by MSA of VP35 sequences of Ebola virus.

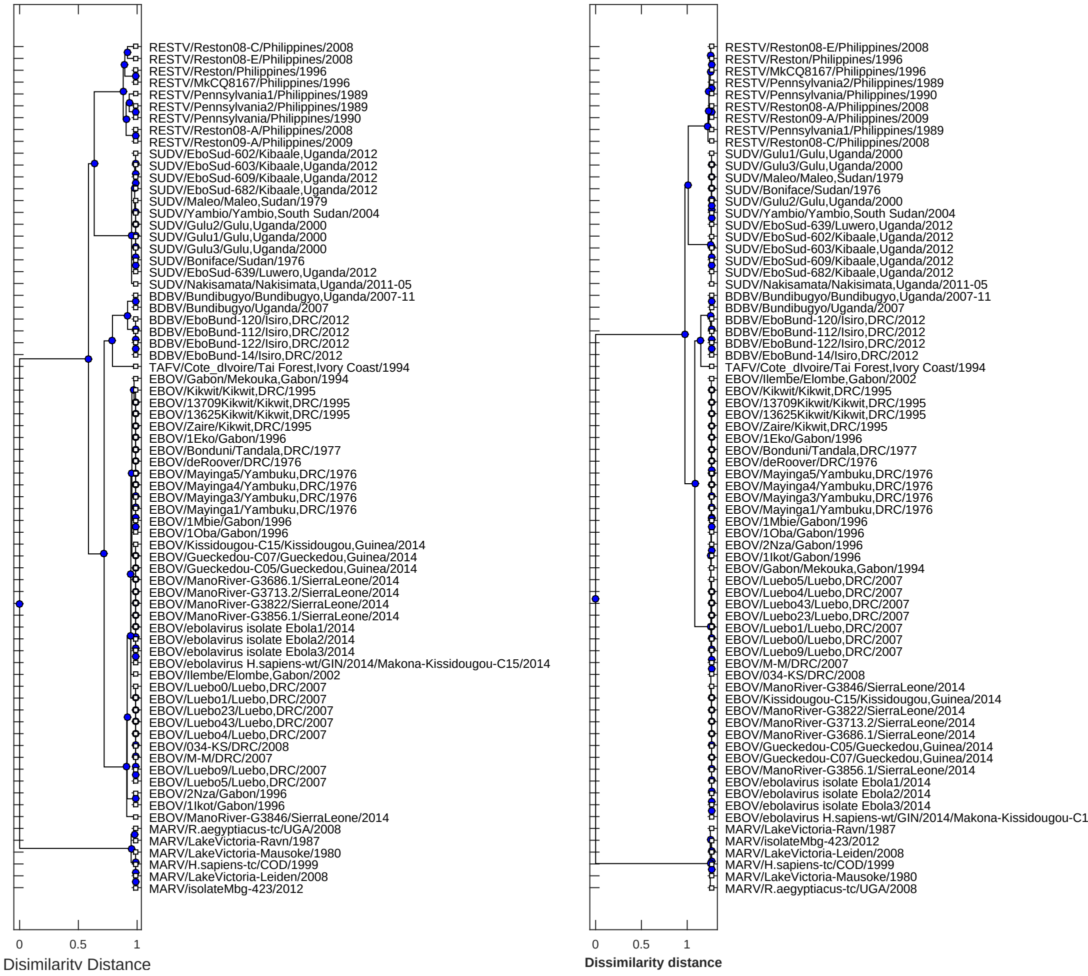

(a) DFT **(b)** MSA

Figure 7. Phylogenetic analysis of Ebola virus. (a) Phylogenetic tree constructed by the DFT distance of VP40 sequences of Ebola virus. (b) Phylogenetic tree constructed by MSA of VP40 sequences of Ebola virus.
